# Release kinetics, static and dynamic water contact angles and FTIR data for tissue inhibitor of matrix metalloprotease-1 (TIMP-1) incorporated in electrospun random DegraPol® fibers and TIMP-1 impact on tenocytes and adipose-derived stem cells proliferation and gene expression data

**DOI:** 10.1016/j.dib.2025.111468

**Published:** 2025-03-19

**Authors:** Julia Rieber, Roger Khalid Niederhauser, Pietro Giovanoli, Johanna Buschmann

**Affiliations:** Division of Plastic Surgery and Hand Surgery, Sternwartstrasse 14, University Hospital Zurich, Zurich 8091, Switzerland

**Keywords:** Tissue inhibitor of matrix metalloprotease-1, Water contact angle, Hydrophobicity, Rabbit Achilles tenocytes, Rabbit adipose-derived stem cells collagen type I, ki67, Tenomodulin, Alkaline phosphatase

## Abstract

A first data set refers to tissue inhibitor of matrix metalloprotease-1 (TIMP-1) protein inclusion into a DegraPol® fibres utilizing emulsion electrospinning and the characterization of the random fibre mesh. Specifically, the release kinetics of the protein from the mesh was studied over 7 days. Moreover, the static and the dynamic water contact angles were determined. Finally, we assessed Fourier-Transformed Infrared Spectra (FTIR spectra) for DegraPol® with and without TIMP-1.

A second data set represents proliferation data obtained with the Alamar Blue Assay, applied on rabbit Achilles tenocytes and rabbit adipose-derived stem cells, when stimulated *in vitro* with 1, 10, and 100 ng/mL TIMP-1 supplementation compared to the corresponding cell culture without TIMP-1 (control). Furthermore, qPCR was performed and *collagen I, ki67, tenomodulin* and *alkaline phosphatase* gene expression data are presented for both cell types *in vitro* stimulated with 1, 10, and 100 ng/mL TIMP-1 supplementation, respectively, and data are presented as manifold induction compared to a TIMP-1-free cell culture medium (control).

Specifications Table

**Table:** 

Subject	Material sciences; genetics; cell biology
Specific subject area	Emulsion electrospun DegraPol® fibre meshes with incorporated TIMP-1 (water-in-oil emulsion) were characterized. Cell culture data were determined for monolayer tenocyte and adipose-derived stem cell (ASCs) cultures. Tenocytes and ASCs were harvested from freshly isolated New Zealand White Rabbit Achilles tendons of three rabbits and from freshly isolated abdominal adipose tissue, respectively. These monolayer cultures were supplemented with the growth factor TIMP-1 at 1, 10 and 100 ng/mL TIMP-1 with 18S as reference gene, while target genes for *collagen I, ki67, tenomodulin* and *alkaline phosphatase* (ALP) was assessed after a 3-day culture. The gene expression was then compared to the same conditions without TIMP-1 supplementation.
Data format	The data consist of raw data and of analysed data (mean and standard deviation of the mean).
Type of data	Excel files of the type *.xlsx* files (data sets with group labels and numbers)
Data collection	**Release kinetics**: We assessed the release kinetics of TIMP-1 into a PBS solution with 0.1 % rabbit serum albumin (RSA) and measured the released TIMP-1 protein by **ELISA kit**. **Water contact angles (WCA)**: Static and dynamic WCA on the material surfaces were measured by a video-based optical contact angle measuring instrument OCA 35 Dataphysics, Germany. **qPCR**: Rabbit Achilles tenocytes were cultured as monolayers and RNA was extracted by an **RNeasy Plus Mini Kit** from Qiagen, Hilden, Germany; followed by reverse transcription with the **iScript Advanced cDNA Synthesis Kit** (Bio-Rad, Cressier, Switzerland). The quantitative real-time PCR reactions were performed with **CFX Connect Real-Time PCR** Detection System (Bio-Rad, Cressier, Switzerland) and **SsoAdvanced SYBR Green Supermix** (Bio-Rad, Cressier, Switzerland).
Data source location	Proliferation data, gene expression, and release kinetic data were collected in Zurich, Switzerland, at the University Hospital Zurich. Water contact angles were determined in Zurich, Switzerland, at the ETH. FTIR spectra were recorded at the ETH Zurich, too.
Data accessibility	**Repository name**: Mendeley Data **Data identification number**: DOI: 10.17632/3cm7sytr3r.1 and DOI: 10.17632/wth9gfzktv.1 **Direct URL** to data: Electrospun biodegradable DegraPol® tubes with tissue inhibitor of matrixmetalloprotease-1 (TIMP-1) protein: Release kinetics, water contact angles and FTIR characterization - Mendeley Data and Impact of tissue inhibitor of matrixmetalloprotease-1 (TIMP-1) protein on the proliferation and gene expression of rabbit Achilles tenocytes and rabbit adipose-derived stem cells *in vitro* - Mendeley Data
Related research article	Fabrication and Characterization of Electrospun DegraPol&reg; Tubes Releasing TIMP-1 Protein to Modulate Tendon Healing Or: 10.3390/ma18030665 [1]

## 1 Value of the Data

• The water contact angle data can be used again for reference tables containing many different polymers with their water contact angles. DegraPol® is a special co-block polymer and its entry may be interesting for polymer scientists.
• The FTIR spectra and data can be reused for comparison with other polyurethane FTIR spectra than DegraPol® polymer.
• The gene expression data can be reused, while comparing other cells than tenocytes or adipose-derived stem cells under supplementation of TIMP-1 protein– for a comparison of their collagen I, ki67, tenomodulin and/or alkaline phosphatase gene expression.
• These data can be reused, if tenocytes or adipose-derived stem cells from other species than rabbits are grown *in vitro* with TIMP-1 for a comparison of our data assessed with rabbit cells.
• If another growth factor or protein than TIMP-1 is used in an Achilles tenocyte culture or in an adipose-derived stem cell culture harvested from rabbit tissue, our gene expression data can be used again for the comparison of TIMP-1 stimulation with that specific growth factor or protein.
• The gene expression data and the proliferation data can be reused in case not TIMP-1, but TIMP-2, TIMP-3 or TIMP-4 are supplemented to rabbit tenocytes or rabbit Achilles tendons; moreover, our data are useful to be compared also to other bioactive molecules that are given to the culture medium of those cells.
• Studies on extracellular matrix remodeling may reuse our data to uncover the mechanistic pathways underlying the activation/deactivation of key target factors where TIMP-1 protein is involved in the corresponding processes.

## 2 Background

**Fig. 1:**
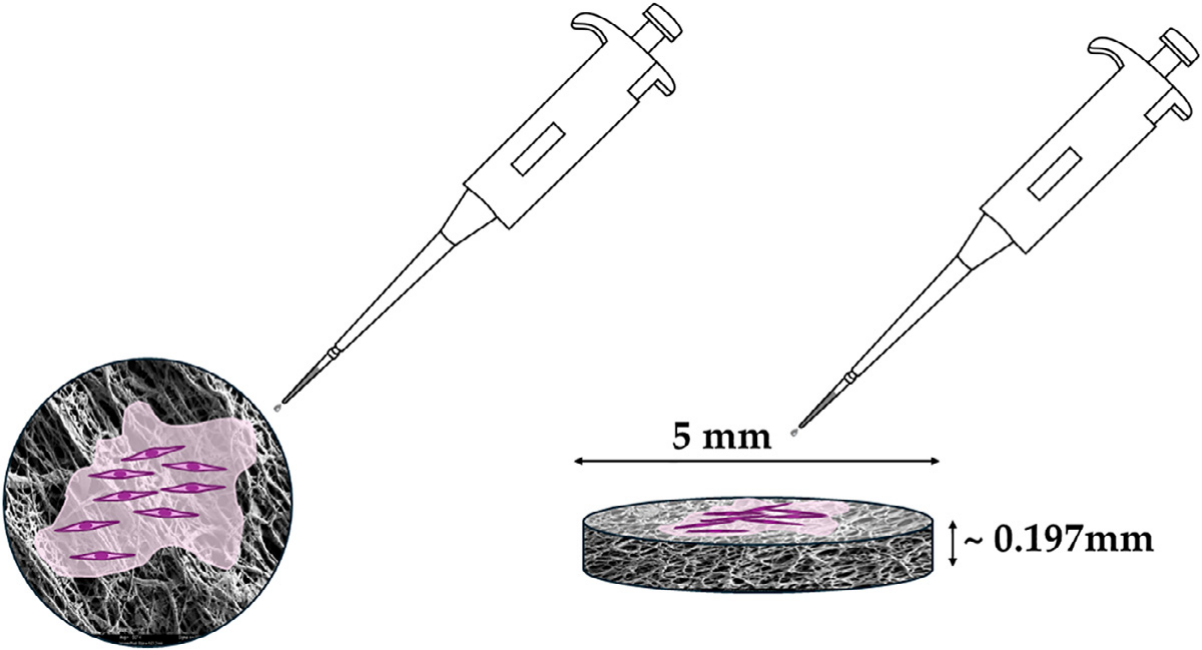
Scanning electron microscopy (SEM) image of electrospun DegraPol® fiber mesh and cells grown on the mesh (purple scheme) (left); size of electrospun disks for this purpose (disks with a diameter of 5 mm and a height of approximately 197 mm, right).

Tendon healing is characterized by an initial short period of inflammation, enduring up to three days. For the next six weeks, there is a highly pronounced cell invasion, and besides pro-inflammatory cells fibroblasts from intrinsic and extrinsic compartments migrate to the laceration site and start with immune/inflammation-dependent tissue remodeling to yield new extracellular matrix (ECM); with collagen III at the beginning to present a template that is later on replaced by collagen I. Finally, between 7 and 12 weeks, sometimes even up to half a year, the modelling and remodeling of the ECM take place, with collagen fiber re-alignment in the direction of force [2]. During remodeling of the tendon tissue, matrix metalloproteases (MMPs) [3] and their inhibitors TIMPs are highly important, because deviations from their optimum ratio during healing might have a non-negligible impact on the outcome [4]. Therefore, delivery devices for factors modulating fibrosis are of high interest in basic research of tendon biology and healing. We here provide data on a novel implant material, based on electrospun DegraPol® fiber meshes, with incorporated TIMP-1 protein and present data on its characterization, including water contact angles, release kinetics and FTIR spectra. In addition, rabbit tenocytes and rabbit adipose-derived stem cells were supplemented with TIMP-1 in different concentrations and the proliferation of those two cells types was measured (Fig. 1). Finally, also the gene expression of these cells under TIMP-1 supplementation is reported here and data on *collagen I, ki67, tenomodulin* and *alkaline phosphatase* are provided.

## 3 Data Description

The data are stored as Microsoft Excel (Microsoft Corporation, Redmond, WA, USA) files (.xlsx files) in the Mendeley Data repository service Electrospun biodegradable DegraPol® tubes with tissue inhibitor of matrixmetalloprotease-1 (TIMP-1) protein: Release kinetics, water contact angles and FTIR characterization - Mendeley Data and Impact of tissue inhibitor of matrixmetalloprotease-1 (TIMP-1) protein on the proliferation and gene expression of rabbit Achilles tenocytes and rabbit adipose-derived stem cells *in vitro* - Mendeley Data.

The first set of data Electrospun biodegradable DegraPol® tubes with tissue inhibitor of matrixmetalloprotease-1 (TIMP-1) protein: Release kinetics, water contact angles and FTIR characterization - Mendeley Data includes **three** excel files, named WCA.xlsx including one sheet; FTIR.xlsx including three sheets and Release.xlsx including one sheet, respectively.

### 3.1 Electrospun DegraPol® Tubes with TIMP-1 Protein: Characterization

#### 3.1.1 File 1 WCA.xlsx

This excel file is open access published in Mendeley Data Electrospun biodegradable DegraPol® tubes with tissue inhibitor of matrixmetalloprotease-1 (TIMP-1) protein: Release kinetics, water contact angles and FTIR characterization - Mendeley Data and contains one sheet, called *Static and dynamic WCA*. In column A, the tube is described, from which the WCAs were measured. In columns B and C, the sample names are noted. In column D, the operator's first name is given, the date of acquisition and the molecular weight of the DegraPol polymer, which is either 80 kDa or 150 kDa. Indication about the location of the experiment, i.e. either outer surface or inner surface, is given in column E. In column F, the category of experiment is noted. In columns G and H, the left and right static WCA is given of replicate 1 in °, while in the subsequent column I, the mean of the left and right static WCA is calculated for replicate 1 in °. Columns J-L and columns N-O represent the same, but for replicates 2 and 2, respectively. Column P gives the mean value of the static water contact angle for all three replicates. Column Q gives the standard deviation of the static water contact angle for the three replicates. Columns R, S and T represent the receding WCA in ° for the left, the right and the mean of left and right, respectively. Column U gives the standard deviation of the receding WCA in °. Column V contains the calculation of the mean and the standard deviation of the receding WCA in ° for all replicates. Columns W to AA give the same information as columns R to V give, but for the advancing WCA in °. Finally, column AB represent the values for the hysteresis, which is the advancing WCA minus the receding WCA in °.

#### 3.1.2 File 2 FTIR.xlsx

**Fig. 2:**
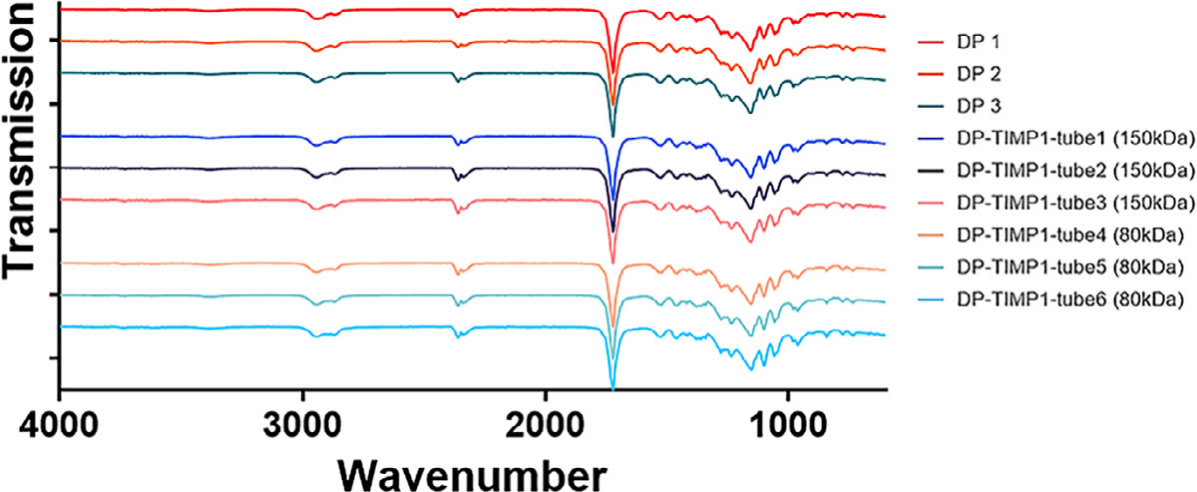
FTIR spectra plotted as transmission (1-absorbance) versus wavenumber (cm-1) of three pure DegraPol tubes (DP1-3); three DegraPol tubes with incorporated TIMP-1 protein and where DP had a 150 kDa molecular weight (DP-TIMP1-tube 1-3 (150kDa)) and three DegraPol tubes with incorporated TIMP-1 protein and where DP had a 80 kDa molecular weight (DP-TIMP1-tube 4-6 (80kDa)).

This excel file is open access published in Mendeley Data Electrospun biodegradable DegraPol® tubes with tissue inhibitor of matrixmetalloprotease-1 (TIMP-1) protein: Release kinetics, water contact angles and FTIR characterization - Mendeley Data and contains three data sheets called *DP(1-3); DP + TIMP1(1-3); and DP + TIMP1(4-6)*. The three sheets refer to the FTIR spectra, where for all three sheets in column A the wavenumber is given in cm^−1^, while in columns B to D the absorbance measured at a specific wavenumber is given for tubes 1, 2 and 3 individually, resulting in the mean of the three tubes, presented in column E. For the first sheet, it is for pure DegraPol (DP) tubes, for the second sheet, it is DP tubes based on a polymer with the molecular weight of 150 kDa and with incorporated TIMP-1, and for the third sheet the same as in the second sheet, however, with the DP polymer exhibiting a molecular weight of 80 kDa. Thus, in the three sheets, the FTIR spectra of three times three tubes data are given. The corresponding spectra were plotted and are shown in Fig. 2.

#### 3.1.3 File 3 Release.xlsx

This excel file is open access published in Mendeley Data Electrospun biodegradable DegraPol® tubes with tissue inhibitor of matrixmetalloprotease-1 (TIMP-1) protein: Release kinetics, water contact angles and FTIR characterization - Mendeley Data and contains one data sheet entitled *Release data*. In column A, the time point is given in days, while in column B, the percentage of the totally released amount at the final time point day 7 is presented (in %). The experiment was repeated 9 times and at each time point, two technical replicates were measured.

### 3.2 Proliferation and Gene Expression Under TIMP-1 Stimulation of Rabbit Tenocytes and Rabbit Adipose-Derived Stem Cells

The second repository called Impact of tissue inhibitor of matrixmetalloprotease-1 (TIMP-1) protein on the proliferation and gene expression of rabbit Achilles tenocytes and rabbit adipose-derived stem cells *in vitro* - Mendeley Data contains **two** Excel files named *Alamar Blue.xlsx and qPCR.xlsx*.

#### 3.2.1 File Alamar Blue.xlsx with One Sheet Called *Alamar Blue*

In the sheet *Alamar Blue*, in column A the percentage of increase between day 1 and day 3 is presented. In column B, information on what cell type was used is presented. Either rabbit Achilles tenocytes were used (tenocytes) or rabbit adipose-derived stem cells were used (ASCs). In the next column C, we provide the concentration of the TIMP-1 protein that was supplemented to the cell culture medium. In columns D and E, the cell number on day 1 and 3 are presented. In column F, the group is given in an abbreviated form, including information on cell type and on TIMP-1 concentration. For example, *Tenos-10* means that tenocytes were used and that the TIMP-1 concentration was 10 ng/mL. Another example: ASCs-1 denotes a cell culture of adipose-derived stem cells under supplementation of 1 ng/mL TIMP-1 protein.

#### 3.2.2 File 2 qPCR.xlsx with six sheets named *27 points arrangement 1, 27 points arrangement 2, 9 points arrangement 1, 9 points arrangement 2, 3 points arrangement 1, 3 points arrangement 2*

The first sheet *27 points arrangement* 1 shows in column A the TIMP-1 concentration under Condition; in column B the gene expression of collagen I in an ASC cell culture, in column C, the ki67 gene expression for ASC cell culture, in column D the tenomodulin gene expression in an ASC culture and in column E, the ALP gene expression in an ASC cell culture. For the next four columns, F to I, the gene expression of these four genes is given in the same order for rabbit tenocytes. In addition, we calculated the mean of the three technical replicates in columns J to Q. Colmuns R to Y represent the same values as columns J to Q, but without formula in the background of each entry. The second sheet denoted as *27 points arrangement 2* presents gene expression data, with column A indicating the TIMP-1 concentration, column B the cell type of the *in vitro* cell culture, column C the number of the rabbit donor from which the cells (both types of cells) were harvested, column D the 2^-DDCT^ value for *collagen I* gene expression, column E the 2^-DDCT^ value for *ki67* gene expression, column F the 2^-DDCT^ value for *tenomodulin* gene expression and column G the corresponding gene expression of *alkaline phosphatase*. Thus, sheets *27 points arrangement 1* and *27 points arrangement 2* show the same data, but differently arranged, which is indicated in the sheet names by *arrangement 1* and *2*, respectively.

The third sheet entitled *9 points arrangement 1* has the TIMP-1 concentration in column A, followed by gene expression of collagen I, ki67, tenomodulin and ALP; for ASC culture (columns B to E) and for tenocytes cell culture (columns F to I), respectively. Here the values of the three technical replicates are shown that have been calculated in the first sheet as described above. The fourth sheet 9 points arrangement 2 is organized as the second sheet, with the difference of presented the mean values of the three technical replicates. Moreover, in column G, a group name is given, with the cell type first and the concentration of TIMP-1 after the hyphen.

In the fifth sheet entitled *3 points arrangement 1*, the columns and content are the same as in the sheet *9 point arrangement 1*, however, the mean of the three experimental replicates (each rabbit donor experiment was done in triplicates) is presented for each donor. Finally, in the sixth sheet entitled *3 points arrangement 2*, the means of the three experimental replicates for each rabbit donor are presented as follows: column A gives the TIMP-1 concentration, column B the cell type, column C the donor and columns D to G the gene expressions for *collagen I, ki67, tenomodulin* and *alkaline phosphatase*, respectively.

## 4 Experimental Design, Materials and Methods

### 4.1 TIMP-1 Water-in-Oil Emulsion Electrospinning

The bilayered scaffolds were fabricated with an in-house electrospinning machine, using DegraPol® polymer and an aqueous TIMP-1 protein solution, according to the protocols previously published [1].

### 4.2 Static and Dynamic Ater Contact Angles

The static and dynamic water contact angles (WCAs) for pure DP and DP emulsion electrospun meshes with TIMP-1 were recorded according to previous protocols [1].

### 4.3 FTIR Spectra

All fourier-transformed infrared spectra were assessed with a Varian 640 Fourier Transform Infrared Spectrometer as previously reported [1].

### 4.4 Release Kinetics

The TIMP-1 protein release kinetics were determined over a period of 7 days, using protocols that have been reported earlier [1].

### 4.5 *In Vitro* rabbit Achilles Tenocyte and Adipose-Derived Stem Cell Culture

Besides rabbit tenocytes that were extracted from Achilles tendons of female New Zealand White rabbits aged between 12 and 16 weeks isolated by cell outgrowth/migration methodology, also rabbit adipose-derived stem cells (ASCs) were harvested from these New Zealand White rabbits (Veterinary license of Canton Zurich, reference number 255/15). A detailed protocol for both cell types as well as assessment of proliferation can be found in the related research article [1]

### 4.6 qPCR

TIMP-1 supplementation to both cell types, i.e. tenocytes and ASCs from three donor rabbits, was performed in different concentrations (1, 10 and 100 ng/mL TIMP-1, respectivelyL) and qPCR was performed for collagen 1, ki67, tenomodulin and alkaline phosphatase genes according to protocols published previously [1]. All primer sequences can be found in the related research article [1].

## Limitations

Gene expression data were obtained only for target genes encoding collagen I, ki67, tenomodulin and alkaline phosphatase; however, other target genes, such as for collagen III, Mohawk or pro-inflammatory markers like IL-6 may would have been interesting as well. Moreover, extracellular matrix (ECM) proteins, such as collagen I and III, fibronectin, laminin, among others, may be interesting to be analysed by Western Blot, because TIMP-1 may affect protein expression of ECM markers.

## Ethics Statement

The rabbit Achilles tenocytes were harvested from rabbits using an animal license approved by the veterinary office of Canton Zurich, Switzerland, having the reference number 255/15.

## CRediT authorship contribution statement

**Julia Rieber:** Conceptualization, Methodology, Data curation, Investigation, Writing – original draft, Writing – review & editing. **Roger Khalid Niederhauser:** Data curation, Investigation, Writing – review & editing. **Pietro Giovanoli:** Supervision, Writing – review & editing. **Johanna Buschmann:** Conceptualization, Supervision, Methodology, Writing – original draft, Writing – review & editing, Funding acquisition, Project administration.

## Data Availability

Mendeley DataElectrospun biodegradable DegraPol® tubes with tissue inhibitor of matrixmetalloprotease-1 (TIMP-1) protein: Release kinetics, water contact angles and FTIR characterization (Original data). Mendeley DataImpact of tissue inhibitor of matrixmetalloprotease-1 (TIMP-1) protein on the proliferation and gene expression of rabbit Achilles tenocytes and rabbit adipose-derived stem cells in vitro - Mendeley D (Original data).
